# Assessing *Listeria monocytogenes* Growth in Artificially Inoculated Sea-Farmed Product—Raw Sea Bass (*Dicentrarchus labrax*) Fillet, Produced in Greece

**DOI:** 10.3390/microorganisms12101970

**Published:** 2024-09-28

**Authors:** Ntina Vasileiadi, Theofania Tsironi, Georgia D. Mandilara

**Affiliations:** 1 Unit of Environmental Microbiology, Laboratory of Infectious Disease Surveillance, Faculty of Public Health Policy, School of Public Health, University of West Attica, 11521 Athens, Greece; elpasofia@gmail.com; 2 Laboratory of Food Process Engineering, Department of Food Science and Human Nutrition, Agricultural University of Athens, 11855 Athens, Greece; ftsironi@aua.gr

**Keywords:** *Listeria monocytogenes*, challenge test, growth potential, sea-farmed product, fish, ready-to-eat food, food safety

## Abstract

*Listeria monocytogenes* (*Lm*) is responsible for listeriosis, a serious foodborne disease, with high hospitalization and mortality rates worldwide. The main cause of listeriosis in humans is the consumption of ready-to-eat (RTE) foods; Commission Regulation (EC) No 2073/2005 establishes microbiological criteria for *Lm* in RTE foods. Raw fish products are widely consumed, e.g., in sushi and various seafood recipes (e.g., carpaccio, sashimi, maki, nigiri, tartare, etc.), but are not subjected to RTE food safety criteria. The aim of our study was to assess the growth potential of *Lm* in raw sea bass fillets obtained from a leading aquaculture company in Greece. In order to assess the growth of *Lm* in raw sea bass fillets, we applied the “challenge test”, a scientific experiment designed to assess the growth of *Lm* within a specific food product under controlled conditions. According to our results, and taking into consideration the health risk for the listeriosis-vulnerable population, raw fish products utilized in the preparation of RTE foods, including sushi and an array of seafood dishes, should be incorporated in the Category of Safety Criteria of Regulation (EC) No 2073/2005 “Ready-to-eat food able to support the growth of *Listeria monocytogenes*”.

## 1. Introduction

Listeriosis, a serious zoonotic infection with high mortality in humans and animals, is caused by *Listeria monocytogenes* (*Lm*). *Lm* is a Gram-positive aerobic and facultative anaerobic bacterium ubiquitous in the environment, including in water, sewage, soil, silage, and feces. It is psychotrophic and exhibits remarkable resistance to diverse environmental conditions such as low pH, low temperature, and high salt concentration. Therefore, it can survive under various food processing procedures such as smoking, drying, salting, and freezing [1,2,3,4]. Due to the ability of *Lm* to form biofilms, it can persist for prolonged periods in the food processing environment, such as siphons, counters, floors, equipment, and drainage filters [5,6]. Without effective prevention and control measures, *Lm* constitutes a potential risk for cross-contamination in the food chain.

Contaminated food, mainly RTE food, is a major route of transmission of *Lm*. RTE food, as defined by Regulation (EC) No 2073/2005, is “food intended by the producer or the manufacturer for direct human consumption without the need for cooking or other processing effective to eliminate or reduce to an acceptable level micro-organisms of concern” [7]. Various RTE foods have been identified as potential carriers of *Lm*, such as smoked and salted fish [8,9,10,11], soft cheese from pasteurized and unpasteurized milk [12,13,14], ice cream [15], salads [16,17], fruits [18,19], pâté [20], and meat and meat products [21,22,23], etc. Listeriosis can manifest in two distinct forms: non-invasive and invasive. While non-invasive listeriosis typically presents with mild, self-limiting symptoms in healthy individuals, invasive listeriosis can pose a severe threat to vulnerable populations. Immunocompromised individuals, the elderly, pregnant women, and infants are particularly susceptible to developing severe and potentially life-threatening infections. Invasive listeriosis can lead to a range of serious adverse outcomes, including abortion, stillbirth, meningitis, sepsis, neurological diseases, and, in severe cases, death [24]. Listeriosis manifests in both sporadic cases and outbreaks.

The dose-response relationship between *Lm* and humans is a complex issue with significant implications for public health. Ongoing scientific investigations seek to clarify the complex interplay of factors influencing this relationship, including host susceptibility, pathogen virulence, food type, consumption quantity, and microbial concentration. Quantitative analyses of *L. monocytogenes* in implicated food items have demonstrated a broad spectrum of contamination levels, ranging from low to high [25,26]. EFSA modeling indicates that 90% of invasive listeriosis cases arise from the consumption of RTE foods harboring microbial counts exceeding 2000 CFU/g [27]. Epidemiological studies have indicated a potential for disease emergence, particularly among vulnerable populations, due to the widespread distribution of food products contaminated with low levels of *Lm* that do not support bacterial growth [28]. Levels below 100 CFU/g at the point of consumption, aligned with legal limits at the end of shelf-life, are generally considered safe, implying a negligible risk of listeriosis for consumers [27]. While it is acknowledged that additional data are required to determine the infective dose and infection probability within vulnerable populations, current knowledge remains limited [29,30].

Based on the European Food Safety Authority and European Centre for Disease Prevention and Control (EFSA-ECDC) report for 2022, the listeriosis notification rate within the European Union/European Economic Area (EU/EEA) was 0.62 cases per 100,000 population [31]. The demographic analysis revealed a high burden on individuals aged 64 and over, with a notification rate of 2.1 cases per 100,000 population in this age group. Hospitalization and fatality rates were substantial, reaching 96% and 18.1% per 100,000 population, respectively. Listeriosis ranked fifth among the most frequently reported zoonoses in the EU during this period. While the overall trend in listeriosis cases between 2018 and 2022 did not present significant fluctuations, 2022 marked a historical high in both the number and rate of reported cases within the EU/EEA. In terms of foodborne outbreaks, 35 incidents were documented, resulting in 296 illnesses, 242 hospitalizations, and 28 fatalities. Among the implicated food items, pig meat, and products thereof, were associated with the highest number of outbreaks (5), followed by fish and fish products (4). Other contributing factors included mixed foods, vegetables and juices, and dairy products.

In Greece, between 2004 and 2023, a total of 266 cases of listeriosis were documented. The average annual incidence rate was calculated to be 1.23 cases per 1,000,000 population. Notably, the years 2015 and 2023 witnessed a surge in listeriosis cases, with the incidence rate reaching 3.0 cases per 1,000,000 population in 2023. A significant proportion of these cases, specifically half, were reported among immunocompromised individuals, pregnant women, and newborns. The overall fatality rate associated with listeriosis in Greece during this period was 24%, with 59 deaths recorded among the 246 cases where the outcome was known [32]. Unpublished data from the National Reference Laboratory (NRL) of Greece for *Lm* demonstrate a notable rise of sporadic human cases in Greece in 2024, with smoked fish involved in most cases. The Hellenic Public Health Organization issued an alert to raise awareness and encourage investigation [33].

All of the above constitute huge concerns and challenges for both the food industry and regulatory public health authorities. European Regulation (EC) No 2073/2005 establishes stringent food safety criteria for specific microorganisms in food products, including *Lm* in RTE foods [7]. Based on the fact that the pH and water activity (a_w_) of the food matrix are crucial for the growth (or not) of *Lm*, Regulation (EC) No 2073/2005 outlines different microbiological criteria based on the food’s ability to support the growth of *Lm*. Four specific factors have been recognized as posing significant limitations for the growth of *Lm*: a product with pH ≤ 4.4; a product with a_w_ ≤ 0.92; a product with a combination of pH ≤ 5.0 and a_w_ ≤ 0.94; and products with a shelf-life of fewer than five days. In these scenarios, the Regulation deems the product safe, as long as *Lm* levels do not exceed 100 colony-forming units per gram (CFU/g) throughout its shelf-life. However, for RTE foods capable of supporting *Lm* growth, stricter measures are necessary. The Regulation allows a food business operator (FBO) to demonstrate that *Lm* will not exceed the 100 CFU/g limit throughout the shelf-life of the product. In the case that an FBO cannot demonstrate control below the 100 CFU/g limit, then the Regulation mandates the FBO test the RTE food for the absence of *Lm* in a 25-g sample. This requirement serves as a public health safeguard; stricter testing for complete absence in a representative sample helps to ensure the safety of the food. Otherwise, the FBO must implement a science-based approach to verify that *Lm* growth remains below the 100 CFU/g limit for the entirety of the product’s shelf-life, through studies such as challenge tests and durability studies. These studies incorporate all stages of food production, processing, and distribution, including retail, and consider reasonably foreseeable conditions of abuse that may occur during the distribution, storage, and consumer use of the product. Several studies have been conducted and published regarding challenge testing and monitoring the growth of *Lm* in many different RTE foods, such as soft cheese, smoked salmon, etc. [34,35,36,37].

RTE fish products are a widely consumed and commercially successful category within the seafood industry. These products, including smoked, canned, cooked, chilled, marinated, pickled, and processed fish, are typically considered safe for consumption without additional preparation. However, RTE fish products have been implicated in multiple strong-evidence listeriosis outbreaks within the European Union in recent years, including in 2022 [31].

Sushi and various seafood recipes (e.g., carpaccio, sashimi, maki, nigiri, tartare, etc.) made from raw fish or fish fillets are on the rise in Europe, adapting to European tastes; these dishes are perceived as a healthy food option, associated with a modern and sophisticated lifestyle. *Lm* has been detected in sushi products in various studies. Factors contributing to the presence of *Lm* in sushi dishes may include raw fish, rice, or other ingredients such as vegetables or sauces [6,38]. Post-harvest contamination is the most common source of *Lm* in raw fish. Cross-contamination can occur during fish handling and processing, that is, during filleting, slicing, or packaging (e.g., contact with contaminated equipment, surfaces, or personnel). Moreover, improper storage conditions, such as inadequate refrigeration, can allow *Lm* to multiply on fish surfaces. From the few investigations that have been carried out so far, it appears that raw fish farmed or harvested in coastal areas or lakes may also be contaminated with *Lm* [39]. While *Lm* has been detected in raw fish, it is generally not considered a ready-to-eat (RTE) food due to the expectation that it will be cooked prior to consumption. However, when raw fish is intended for use in sushi recipes, *Lm* poses a significant public health risk.

This study aimed to investigate the growth potential of *Lm* in raw sea bass fillets obtained from a Greek sea farm, with the goal of evaluating the safety of consuming raw fish for RTE purposes without adhering to established regulations and safety standards. Sea bass, renowned for its mild flavor and firm texture, is a popular choice for sushi and other raw seafood preparations. In recent years, raw sea bass has been introduced as a key ingredient in certain seafood sushi products [40]. Moreover, sea bass, particularly the European sea bass (*Dicentrarchus labrax*), is a popular fish species commonly farmed in large-scale marine aquaculture operations.

## 2. Materials and Methods

In order to assess the growth of *Lm* in raw sea bass fillets, we applied the challenge test; the aforementioned test in the context of *Lm* and food safety is a scientific experiment designed to assess the growth of *Lm* within a specific food product under controlled conditions. The whole procedure was based on the latest version of the “EURL *Lm* Technical Guidance Document on challenge tests and durability studies for assessing shelf-life of RTE foods related to *Lm*” [41] (the current citation “EURL *Lm* TGD”, 2021, is valid throughout the whole text) and the ISO 20976-1:2019 [42].

### 2.1. Food Product Contamination Level-Storage Temperatures

**Figure 1:**
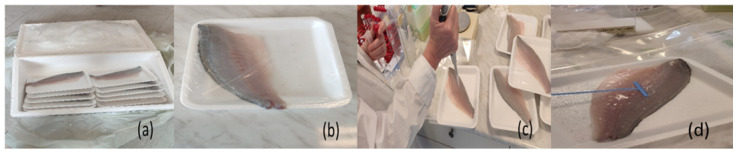
When delivered, the fish fillet trays were packaged in polystyrene boxes with a large ice pack inside and a protective film between the product and the ice pack. There were 10 trays in each polystyrene box (**a**). Fish fillets were packed in expanded polystyrene trays and wrapped with stretch film. The net weight of each fillet was from 90 g to 110 g (**b**). In order to inoculate the raw sea bass fillet samples, the stretch film was removed, fish surface contamination and spread of *Lm* inoculum were performed, and finally, the trays were repacked with the protective film (**c**,**d**).

The studied food product was raw sea bass (*Dicentrarchus labrax*) fillets, obtained from a leading aquaculture company in Greece. Fish fillets are packed in expanded polystyrene trays and wrapped with stretch film (forwarded to supermarkets in this exact form). The net weight of each fillet is from 90 g to 110 g. Each tray contains one fish fillet (Figure 1b). The commercial shelf-life of the product is 4 days at 4 °C. Raw fish fillets have undergone no processing and there is no addition of preservatives.

Before starting our research, several meetings were held with the executives of the fish production company to obtain the necessary information on the product characteristics, production conditions and processes, shelf-life, product packaging procedure, temperatures during production, and transportation, at both the retail and the consumer level. Based on all the above information, we designed the research protocol. We then performed several test experiments in order to finalize the protocol to be implemented.

We received the product at the laboratory from the company on the first day of production-filleting. In our research, this day was designated as “Day 0”; “Day End” is the 8th final day of our study, even if the product’s shelf-life is 4 days. We decided to gather information on all parameters (microbiological and physico-chemical) beyond the shelf-life of the product in order to get more comprehensive data regarding the growth of *Lm*. The fish fillet trays, before being delivered, were packaged in polystyrene boxes with a large ice pack inside and a protective film between the product and the ice pack. There were 10 trays in each polystyrene box (Figure 1a). Each Batch contained approximately 26 fish tray samples (20 samples for the study tests and 6 additional samples in case of failure or mishandling). Six distinct Batches, comprising a total of 156 samples, were delivered to the laboratory by the company. These Batches represented production from six separate days. The temperature of the product at the time of receipt was from 0.5 °C to 2 °C.

Artificial contamination with *Lm* 10 CFU/g (“very low” contamination level) and with *Lm* 50 CFU/g (“low” contamination level) were performed. For each applied level of contamination, three different Batches from three different production days were used; three Batches for Experiment 1 (Batches 1,2,3 with *Lm* 10 CFU/g) and three Batches for Experiment 2 (Batches 4,5,6 with *Lm* 50 CFU/g).

The protocol and the temperature parameters that we applied in our study were agreed upon with the company and were based on cold chain conditions, which should mimic the actual conditions of product storage on all levels, from production to consumer level: two days at 2 °C (to mimic the storage conditions at production and transportation levels) [41], four days at 4 °C (to mimic storage conditions at the retail level) [43], and the last two days at 10 °C (to mimic storage conditions at the consumer level) [44,45].

### 2.2. Inoculum

A mix of three different strains of *Lm*, isolated from the fish products and related environments, was used in order to avoid the bias associated with the use of only one *Lm* strain.

The used *Lm* strains were the following:

1. Strain reference: 03EB425*LM*, molecular serotype IIa, clonal complex 101, Sequence Type 775, isolated from fish; 2. Strain reference: 11CEB245*LM*, molecular serotype IIa, clonal complex 193, Sequence Type 193, isolated from fish; 3. Strain reference: 12MOB049*LM*, molecular serotype IIb, clonal complex 3, Sequence Type 3, isolated from the food industrial environment.

Before receiving each Batch of fish fillet, in order to prepare the *Lm* inoculum, the cultures of each of the above *Lm* strains were prepared and *Lm* was enumerated. A mixed culture was prepared by mixing the above three *Lm* strains in the same concentration. From the mixed culture, using decimal dilutions with physiological water, the inoculum was prepared in a volume sufficient to inoculate all the “Test Units”. The *Lm* concentration in the inoculum was enumerated according to ISO 11290-2 [46].

The target contamination of *Lm* level for the three Batches of Experiment 1 was “very low”, 10 CFU/g. This is the level at which, on the first day of production of the product, *Lm* is likely not to be detected using *Lm* enumeration method ISO 11290-2 or *Lm* detection method ISO 11290-1 [47].

The inoculum concentrations of *Lm* for the three Batches of Experiment 1 were: for Batch 1, 1.5 × 10^4^ CFU/mL; for Batch 2, 2.1 × 10^4^ CFU/mL; for Batch 3, 1.8 × 10^4^ CFU/mL.

The target contamination of *Lm* level for the three Batches of Experiment 2 was “low”, 50 CFU/g, that is, below the legal food safety limit of Regulation (EC) No 2073/2005.

The inoculum concentrations of *Lm* for the three Batches of Experiment 2 were: for Batch 4, 1.7 × 10^4^ CFU/mL; for Batch 5, 2.2 × 10^4^ CFU/mL; for Batch 6, 1.5 × 10^4^ CFU/mL.

### 2.3. Inoculation-Contamination

Seven samples called “Test Units” were inoculated with *Lm* on the day the samples were received in the laboratory, that is, “Day 0”. In order to inoculate the raw sea bass fillet samples, we removed the stretch film from the tray with the fish fillet, performed surface contamination, spread *Lm* inoculum on the sample, and put the protective film back on the tray (Figure 1c,d).

The volume of the inoculum per sample—“Test Unit”—should not have exceeded 1% of the mass of the sample, following the “EURL *Lm* TGD” [41]. For each Batch, additional samples, called “Control Units”, were inoculated with physiological water at the same volume as the “Test Units” were inoculated with *Lm* inoculum. Moreover, “Food Control Samples” (samples not subjected to any preparation, without any inoculation) were also used (Table 1).

### 2.4. Experimental Design

On “Day 0”, to verify the absence of *Lm*, detection analyses were conducted on five “Food Control Samples” per Batch. These same samples were also subjected to pH, water activity (a_w_), sodium chloride (NaCl) content, and fat content assessments. Additionally, background microbial flora analysis, including mesophilic aerobic count and *Pseudomonas* spp. count, was performed to evaluate intra-batch variability.

To assess the influence of changes in food composition on physicochemical parameters, seven “Control Units” per Batch were subjected to measurements of pH, water activity (a_w_), sodium chloride (% NaCl), fat content, mesophilic aerobic count, and *Pseudomonas* spp. count. These measurements were conducted on “Day 0” and on the 4th, 6th, and 8th days (Table 1). “Control Units”, designed to replicate the physicochemical conditions of inoculated “Test Units”, were used to evaluate any effects arising from variations in the actual food composition. Additionally, one “Control Unit” per Batch was employed to monitor storage temperatures. A thermal data logger (Elitech RC-5) was placed within a designated “Control Unit”, situated within the same incubator as the “Test Units”, to record temperature values throughout the experimental period.

For three of the seven “Test Units”, enumeration of *Lm* was conducted immediately (“Day 0”). The remaining inoculated “Test Units” were stored at 2 °C for two days to simulate production and transportation conditions. Interim *Lm* enumeration was performed on “Test Units”: after two days (“Day 2”) at 2 °C, after a further 2 days (till “Day 4”) at 4 °C (simulating retail storage), after an additional 2 days (“Day 6”) at 4 °C, and finally, after another 2 days at 10 °C (simulating consumer storage) on the last day (“Day End”).

Although the product’s stated shelf-life was four days, the challenge test period was extended to eight days to evaluate the product’s actual shelf-life, assess *Lm* growth, and monitor changes in physicochemical characteristics and associated microbial flora.

For all the above analyses of microbiological criteria testing, ISO methods were applied [46,47,48,49]. Fat, salt in the aqueous phase, a_w_, and pH values were determined according to AOAC procedures [50].

### 2.5. Data Analysis

For each Batch inoculated with *Lm*, the growth potential (Δ) was calculated according to the formula: Δ = log_max_ − log_i_, where log_max_ is the highest value of the *Lm* enumeration obtained from at least 4 sampling points (excluding the sampling at “Day 0”), wherein one “Test Unit” is analyzed per sampling point. The log_i_ is the mean value of the 3 “Test Units” analyzed at “Day 0”. The Growth Potential retained among all tested Batches is the highest obtained (Δ) value [41]. If (Δ) is lower or equal to the limit of 0.5 log_10_, then it is assumed that the food is not able to support the growth of *Lm* (Category 1.3 of Regulation (EC) No 2073/2005). If (Δ) is higher than the limit of 0.5 log_10_, then it is assumed that the food is able to support the growth of *Lm* (Category 1.2 of Regulation (EC) No 2073/2005) [7].

Analysis of variance (two-factor ANOVA) at a significance level of 95% was applied for the analysis of the studied physicochemical and microbiological parameters for all sample series (XLSTAT 2023.1.1, https://www.xlstat.com/en/; accesed on 25 August 2024).

## 3. Results

### 3.1. “Food Control Samples”—Samples Not Subjected to Any Inoculation

After receiving the samples of each Batch in the laboratory, on “Day 0”, the absence of *Lm* in the five “Food Control Samples” was confirmed: “*Lm* was not detected” in 25 g (*n* = 5, for each Batch).

Physico-chemical characteristics of the “Food Control Samples” on “Day 0” were more or less stable in the three Batches of Experiment 1 and three Batches of Experiment 2, representing six different production dates (*p* > 0.05). The measurement results were close to the optimal conditions that favor the growth of *Lm* (pH ≈ 7, a_w_ ≈ 0.99) (Table S1).

### 3.2. Assessing of Growth of Listeria monocytogenes—Growth Potential

#### 3.2.1. “Test Units”—Samples Inoculated with *Lm* 10 CFU/g

The growth of *Lm* in the “Test Units” inoculated with 10 CFU/g *Lm*: three Batches of Experiment 1 (1, 2, 3), from “Day 0” to “Day 2”, at the storage temperature of 2 °C, and from “Day 2” to “Day 4” at the storage temperature of 4 °C, were approximately at the same levels. On the contrary, the % concentration increase of growth of *Lm* from “Day 4” to “Day 6” at the storage temperature of 4 °C was 63.09% for Batch 1, 71.81% for Batch 2, and 52.75% for Batch 3. Τhe % concentration increase of growth of *Lm* in the three Batches of Experiment 1 from “Day 6” to “Day 8” at the storage temperature 10 °C was 33.08% for Batch 1, 31.13% for Batch 2, and 35.68% for Batch 3. Τhe % concentration increase of growth of *Lm* from “Day 4” (end of shelf-life) to “Day 8” (end of our study) was 117.04% for Batch 1, 125.30% for Batch 2, and 107.25% for Batch 3; in total, the % concentration increase of *Lm* growth from “Day 0” to “Day 8” was 247.71% for Batch 1, 282.78% for Batch 2, and 232.03% for Batch 3 (Table 2).

In the first days after inoculation of “Test Units” with 10 CFU/g in the three Batches of Experiment 1, the growth of *Lm* was very low; therefore, the Growth Potential (Δ) could not be calculated.

#### 3.2.2. “Test Units”—Samples Inoculated with *Lm*, 50 CFU/g

In order to be possible to estimate the Growth Potential of *Lm* in the studied fish product, the “Test Units” of the three Batches of Experiment 2 (4, 5, 6) of raw sea bass fillets were inoculated with *Lm* 50 CFU/g.

In the fish fillets inoculated with 50 CFU/g, from “Day 0” to “Day 2”, the growth of *Lm* had exceeded the safety limit of 100 CFU/g established by (EC) Regulation 2073/2005 [7]. From “Day 2” to “Day 4”, the growth rates of *Lm* doubled. From “Day 4” to “Day 6”, there was almost 1 log_10_ of *Lm* growth, while from “Day 6” to “Day 8”, there was about an additional 1-log_10_ increase (Table 3).

According to the “EURL *Lm* TGD”, the growth potential of *Lm* was determined, (Δ) = 2.63 log_10_ CFU/g, that is, > 0.5 log_10_ CFU/g. The standard deviation (SD) between three replicates at “Day 0” for Batch 4 is 0.05, for Batch 5 is 0.08, and for Batch 6 is 0.14. The above SD is lower than <0.3 log_10_ CFU/g, which means that *Lm* inoculum was homogeneously distributed in all units of food, based on the “EURL *Lm* TGD” (Table 4).

### 3.3. Physico-Chemical Analysis of “Control Units”—Samples Inoculated with Physiological Water in the Same Volume of the Lm Inoculum to “Test Units”

Physico-chemical measurements performed on the “Control Units” of the three Batches of Experiment 1 and the three Batches of Experiment 2 at “Day 0”, “Day 6”, and “Day 8”—“Day End”, as presented in Tables S3 and S4. The pH, a_w_, and % NaCl values from “Day 0” through “Day 8” did not present significant changes (*p* > 0.05) in all six Batches of Experiment 1 and Experiment 2 during the studied period. Also, no significant differences were observed between the three Batches of Experiment 1 of fish fillets and the three Batches of Experiment 2 regarding physico-chemical analyses (*p* > 0.05). The measurement results were close to the optimal conditions that favor the growth of *Lm* (pH ≈ 7, a_w_ ≈ 0.99). The fat content measurements on “Day 0” in all samples of all 6 Batches were similar (Tables S3 and S4).

### 3.4. Microbiological Analysis

According to our results, at “Day 0”, in all of the samples (“Control Unit” samples, “Food Control” samples, and “Test Units” samples, as described in Table 1) of all six Batches of Experiments 1 and 2, (Tables S5–S9), there is no statistically significant difference in the measurements of mesophilic aerobic count and *Pseudomonas* spp. (*p* > 0.05); the mean colony count per gram of food product for the total mesophilic flora was 3350 CFU/g (range 1100–8500 CFU/g), and for *Pseudomonas* spp. was 2800 CFU/g (range 1100–7300 CFU/g). At “Day 4”, which was the end-of-shelf-life of the fresh fish fillet, both microbiological parameters significantly increased (*p* < 0.05) in all of the samples of all six Batches (no significant differences were observed between different Batches, *p* > 0.05); the mean colony count per gram of food product for the total mesophilic flora was 1.0 × 10^6^ CFU/g (range 0.3–4.5 × 10^6^ CFU/g), and for *Pseudomonas* spp. was 0.75 × 10^6^ CFU/g (range 0.2–2 × 10^6^ CFU/g). At “Day 8”, concentrations experienced a further thousand-fold increase. No significant differences were observed between different Batches (*p* > 0.05), in both the mesophilic aerobic count and *Pseudomonas* spp. in all tested samples.

## 4. Discussion

*Listeria monocytogenes* is a foodborne pathogen capable of causing severe illness, particularly in vulnerable populations such as pregnant women, newborns, the elderly, and immunocompromised individuals. RTE foods, which require no further cooking before consumption, are of particular concern due to their potential for *Lm* contamination. The presence of *Lm* in RTE foods poses a significant public health risk. Outbreaks associated with these products can result in widespread illness, hospitalization, and even fatalities. To mitigate this risk, rigorous food safety measures are essential throughout the food production chain [51].

The production of numerous RTE products incorporates various methodologies designed to inhibit the growth of *Lm*. These strategies encompass the manipulation of intrinsic factors, (such as reducing water activity through processes such as salting, cooking, or drying, and modifying pH levels via fermentation or acidification), the use of various protective packages (e.g., under vacuum, modified atmosphere packaging), or the addition of various protective substances and inhibitors. Collectively, these interventions effectively limit the proliferation of *Lm* within the finished product, before being released to the consumers [52].

Raw fish, despite its common use in sushi-like preparations at restaurants and in homemade recipes, is categorically distinct from RTE foods and is therefore exempt from the regulatory framework outlined in Regulation (EC) No 2073/2005. This regulatory divergence stems from the fundamental distinction between raw to-be-cooked and RTE products, with the latter undergoing processes designed to eliminate or significantly reduce microbial contamination. It has also been documented that raw fish may be contaminated with *Lm*. While the occurrence is generally lower compared to other food products, the potential for contamination exists and poses a significant public health risk. It is well-established that the water activity (a_w_) and pH of raw fish support the growth of *Lm*. However, given that the shelf-life of the specific fish fillet we studied is 4 days, if it would be considered as RTE, it would be classified, according to Regulation 2073/2005, as a food that “does not support the growth of *Lm*”, since its shelf-life is <5 days [7]. The objective of our study was to determine the extent of *Lm* growth within and beyond the shelf-life of raw fish packed and sold in trays that had been inoculated with “very low” and “low” levels of *Lm* and to assess the expiration date of the particular product. In essence, we sought to evaluate the safety of the assumption that this product, when consumed raw, does not pose a public health risk. In order to satisfy the aforementioned concern, we performed a challenge test, as proposed by the “EURL Lm TGD” [41].

### 4.1. “Food Control Samples” and “Control Units”

“Food Control Samples” serve as a critical component of *Lm* challenge tests. Their primary purpose is to establish a baseline microbiological profile and physico-chemical characteristics of the food product prior to the introduction of the studied microorganism. By analyzing “Food Control Samples”, the absence of *Lm* in the product before inoculation is ensured, the product’s intrinsic properties are evaluated (e.g., pH, water-activity a_w_), which can influence the growth potential of *Lm*, and a reference point is provided, in order to enable comparison of microbiological and physico-chemical parameters between the “Test Unit” samples and the uninoculated “Food Control Samples”. “Control Units” are used to track changes in pH, a_w_, and other relevant physico-chemical properties of the food product over time. This helps to confirm that these parameters remain stable throughout the challenge test, ensuring that any observed growth or inhibition of *Lm* is due to the presence or absence of the pathogen, rather than changes in the product itself. According to our results, no *Lm* was detected in any of the six Batches of the studied fish fillet at “Day 0”; physico-chemical parameters remained stable for all six Batches and for “Food Control Samples” and “Control Units” throughout the whole challenge test period (till “Day 8”), close to the optimal conditions that favor the growth of *Lm* (pH ≈ 7, a_w_ ≈ 0.99).

### 4.2. Mesophilic Aerobic Count and Pseudomonas spp.

*Pseudomonas* species are ubiquitous in the aquatic environment and are commonly associated with fish spoilage. Their presence and growth can indicate compromised hygiene practices during processing or storage. Monitoring *Pseudomonas* spp. levels, along with the mesophilic aerobic count, during the challenge test served as a proxy for evaluating the overall microbial load and the effectiveness of sanitation measures. Assessing the native microbial population provides insights into potential competitive or inhibitory effects on *Lm*; the presence of specific microorganisms can influence product pH and a_w_, which in turn impacts *Lm* growth. According to our results (Tables S5–S8), background microbial flora harbored in the product at “Day 0” seems to increase for both microbiological criteria tested in all tested Batches. Moreover, the natural acidification due to the progressive growth of “background” microbial flora during the storage of fish fillets did not cause a decline in pH level capable of suppressing *Lm* growth. Furthermore, despite an elevated concentration of indigenous microflora and potential antagonistic interactions, *Lm* proliferation was not inhibited.

### 4.3. Listeria Monocytogenes Growth

In Batches contaminated with “very low” *Lm* concentration (10 CFU/g, the pathogen was enumerated at the end of shelf-life (4 days) and was <50 CFU/g (Table S2). At “Day 6”, however, *Lm* concentration was >100 CFU/g. Therefore, the shelf-life of the above product, having specific characteristics of pH and a_w_, which support the growth of *Lm,* and in a particular packaging type, cannot be more than 4 days. On “Day 0”, despite the presence of the pathogen in the fish matrix due to our inoculation, either no colonies or 1–3 colonies were detected on the culture medium plate (Table S2). This implies that the “raw fish food sample” may be considered safe; however, *Lm*, especially in a food matrix with pH and a_w_ levels that favor its growth, can reach dangerous concentrations, particularly for the vulnerable population. It is important to note that only a very small portion of every food Batch is inspected during production or on the shelf. Furthermore, very low concentrations of a pathogen within a food sample are highly likely to evade colony counting methods, resulting in the detection of no colonies on a plate; not detecting *Lm* in 25 g in five samples (*n* = 5) of one Batch during food production, as (EC) Regulation 2073/2005 requires, does not ensure the absence of *Lm* in a whole Batch. Moreover, a very low population may not be detected by an analytical laboratory; in our own study, after inoculation with *Lm* 10 CFU/g in two of the nine samples of the three Batches of Experiment 1, we calculated *Lm* < 10 CFU/g, though knowing that we had contaminated the samples (Table S2). Finally, it should be noted that official food inspections at the retail level are not always conducted as frequently as they should be due to a shortage of resources, personnel, and other constraints. Collectively, all the above constitute a great concern for the presence of *Lm* in raw fish to be consumed as RTE food, which warrants serious attention from the food industry and public health authorities.

In Batches contaminated with “low” *Lm* concentration (50 CFU/g), even on the 2nd day of contamination, *Lm* was enumerated >100 CFU/g (Table 3). The calculated growth potential of the studied product—raw sea bass fillets, packed in expanded polystyrene trays, wrapped with stretch film—was (Δ) = 2.63 log_10_ CFU/g, indicating that this certain food product supports *Lm* growth; this finding was expected based on the characteristics of the pH and a_w_.

The concern of the fish company and ours was whether the particular product in a particular packaging type is safe for public health until the end of its shelf-life. According to our results, it may be considered that raw fish used in the preparation of RTE foods, such as sushi and various seafood dishes (e.g., carpaccio, sashimi, maki, nigari, tartare, etc.) consumed without any processing, should be integrated into the category of “Ready-to-eat food able to support the growth of *Listeria monocytogenes*” and classified in food category 1.2 in the (EC) Regulation 2073/2005. Particular emphasis should be placed on vulnerable populations, where listeriosis can be fatal even at low levels of the pathogen in food. United States of America has adopted a zero-tolerance policy for *Lm* in RTE foods. Given the increasing number of listeriosis cases and outbreaks caused by the pathogen, the European legislation on safety criteria for RTE foods concerning *Lm* may need to be reassessed.

## 5. Conclusions

Our findings suggest that a reassessment is warranted regarding the categorization of raw fish, employed in the production of RTE foods such as sushi and various seafood preparations; consideration should be given to classifying these raw fish within the category of “Ready-to-eat food able to support the growth of *Listeria monocytogenes*”.

Challenge testing is a critical component of a comprehensive *Lm* control program for FBOs; it provides essential information for mitigating the risk of *Lm* contamination in RTE foods. Challenge tests provide scientific evidence to support the efficacy of food preservation and processing methods in controlling *Lm* growth. Moreover, challenge testing can be used to evaluate the impact of product formulation, packaging, and processing changes on *Lm* growth; thus, FBOs can determine an accurate number of days of shelf-life of the food product. Many regulatory authorities require or recommend challenge testing as part of a robust food safety program. By conducting challenge tests, FBOs can demonstrate a commitment to food safety and proactive steps to protect consumers from the risk of listeriosis. Appropriate processes and subsequent storage practices can guarantee the safety of their products. Also, they must evaluate the results of challenge tests for every single different food product they produce and interpret them in the most correct way.

It is critically important for FBOs to adhere to food safety procedures for *Lm*, including proper storage temperatures, good hygiene practices, and prevention of cross-contamination between raw and RTE foods; consequently, they ensure consumer protection from a potentially life-threatening illness and also conserve their brand reputation and avoid financial losses.

## Figures and Tables

**Table 1:** Total number of samples used for assessing the growth potential in raw sea bass fillets packed in expanded polystyrene trays, according to the “EURL *Lm* TGD”.

Type of Units	Type of Analysis	Number of Units and Date of Analysis per Batch	
**Test Units**	Enumeration of *Lm*	**7**	3 Test Units at “Day 0”, 1 Test Unit at 3 intermediate dates, and 1 at “Day End”
	Enumeration of the associated microflora	**4**	Enumeration of the associated microflora in 4 of the above 7 Test Units: 1 at “Day 0”, 1 at “Day 4”, 1 at “Day 6”, and 1 at “Day 8”
**Food Control Samples**	Detection of *Lm*	**5**	5 at “Day 0”
	Measurement of physico-chemical characteristics		
	Enumeration of the associated microflora	1	Enumeration of the associated microflora in 1 sample of the above 5 Food Control Samples at “Day 0”
**Control Units**	Measurement of physico-chemical characteristics	**7**	2 at “Day 0”, 2 at “Day 6”, and 2 at “Day 8–Day End” For Enumeration of the associated microflora, 1 more sample at “Day 4”
	Enumeration of the associated microflora		
	Temperature control	**1**	Throughout the test
**Total number of samples tested per Batch** **Total number of analyzed samples**		**20** 20 × 6 * = 120	

* In total, six Batches of fish product were analyzed: three Batches of Experiment 1 (Batches 1,2,3) were inoculated with *Lm* 10 CFU/g and three Batches of Experiment 2 (Batches 4,5,6) were inoculated with *Lm* 50 CFU/g.

**Table 2:** The *Lm* concentration (log_10_ CFU/g) in the “Test Units” of the three Batches of Experiment 1 inoculated with *Lm* 10 CFU/g and % concentration increase of *Lm* growth in the raw sea bass fillets.

	*Lm* (log_10_ CFU/g)				*Lm* % Concentration Increase		
Day (Storage Temperature)	Batch 1	Batch 2	Batch 3		Batch 1	Batch 2	Batch 3
Day 0* (2 °C)	1.00	1.00	1.00				
Day 4 (4 °C)	1.60	1.70	1.60	% of *Lm* growth from Day 0* to Day 4	60.21	69.90	60.21
Day 6 (4 °C)	2.61	2.92	2.45	% of *Lm* growth from Day 4 to Day 6	63.09	71.81	52.75
Day 8 (10 °C)	3.48	3.83	3.32	% of *Lm* growth from Day 6 to Day 8	33.08	31.13	35.68
				% of *Lm* growth from Day 4 to Day 8	117.04	125.30	107.25
*Lm* % concentration increase from “Day 0*” to Day 8, “Day End”					247.71%	282.78%	232.03%

Day 0*—Since the samples were inoculated with a population of 10 CFU/g and the logarithm of 10 is 1, we use this logarithm to be able to calculate the % concentration increase of *Lm* from “Day 0” to “Day 4” and from “Day 0” to “Day 8” (“Day End”).

**Table 3:** The *Lm* concentration (log_10_ CFU/g) in the “Test Units” of the three Batches of Experiment 2 inoculated with *Lm* 50 CFU/g and % concentration increase of *Lm* growth in the raw sea bass fillets.

	*Lm* (log_10_ CFU/g)			*Lm* % Concentration Increase			
Day (Storage Temperature)	Batch 4	Batch 5	Batch 6		Batch 4	Batch 5	Batch 6
“Day 0”	1.75	1.83	1.75				
“Day 2” (2 °C)	2.15	2.16	2.00	% of *Lm* growth from Day 0 to Day 2	22.86	18.03	14.29
“Day 4” (4 °C)	2.57	2.85	2.34	% of *Lm* growth from Day 2 to Day 4	19.53	31.94	17.00
“Day 6” (4 °C)	3.37	3.46	3.04	% of *Lm* growth from Day 4 to Day 6	31.13	21.40	29.91
“Day 8” (10 °C)	4.16	4.46	4.11	% of *Lm* growth from Day 6 to Day 8	23.44	28.90	35.20
*Lm* % concentration increase from “Day 0” to Day 8, “Day End”					137.71%	143.72%	134.86%

**Table 4:** Growth potential of *Lm* in the three Batches of Experiment 2, raw sea bass fillets inoculated with 50 CFU/g. The Growth potential was calculated according to the last version of “EURL *Lm* TGD”.

Growth Potential (Δ) *Lm* log_10_ CFU/g in Sea Bass Fillets Inoculated with 50 CFU/g			
	Batch 4	Batch 5	Batch 6
**“Day 0”** (2 °C)	1.70	1.90	1.60
	1.78	1.85	1.88
	1.78	1.74	1.78
**Mean at “Day 0” (Average)**	**1.75**	**1.83**	**1.75**
**Standard deviation (SD) of the 3 Test Units at “Day 0”**	**0.05**	**0.08**	**0.14**
**Day 2** (4 °C)	2.15	2.16	2.00
**Day 4** (4 °C)	2.57	2.85	2.34
**Day 6** (10 °C)	3.37	3.46	3.04
**Day 8 “Day End”**	4.16	4.46	4.11
Growth potential of *Lm* for each Batch (Δ) Batch = log_max_ − log_i_ (initial, at Day 0) log_10_ CFU/g	Mean at Day 0 = 1.75 ±SD = 0.05 **Δ = 4.16 − 1.75 = 2.41**	Mean at Day 0 = 1.83 ±SD = 0.08 **Δ = 4.46 − 1.83 = 2.63**	Mean at Day 0 = 1.75 ±SD = 0.14 **Δ = 4.11 − 1.75 = 2.36**
Growth potential (Δ) is the highest value of *Lm* log_10_ CFU/g among the 3 Batches of raw sea bass fillets	**2.63**	Δ > 0.5 log10 CFU/g. So, the studied sea bass fillets are “ready-to-eat food able to support the growth of *L. monocytogenes*” and are classified in food category 1.2 in the (EC) Regulation 2073/2005	

## Data Availability

The original contributions presented in the study are included in the article/Supplementary Material, further inquiries can be directed to the corresponding author.

## Supplementary Materials

The following supporting information can be downloaded at: https://www.mdpi.com/article/10.3390/microorganisms12101970/s1. Table S1. Physico-chemical characteristics of the five “Food Control Samples” of each Batch of Experiment 1 (Batch 1,2,3) and Experiment 2 (Batch 4,5,6) of raw sea bass fillets from a sea-farmed product company, produced in Greece; Table S2: Growth of *Lm* in the “Test Units” of the three Batches of Experiment 1 of raw sea bass fillets from a sea-farmed product company, produced in Greece. The raw sea bass fillets were inoculated with *Lm* 10 CFU/g; Table S3: Physico-chemical characteristics of the “Control Units” of the three Batches of Experiment 1 of raw sea bass fillets inoculated with physiological water in the same volume as the 10 CFU/g *Lm* inoculum at “Day 0”, at “Day 6”, and at Day 8, “Day-End”; Table S4: Physico-chemical characteristics of the “Control Units” of the three Batches of Experiment 2 of raw sea bass fillets inoculated with physiological water in the same volume as the 50 CFU/g *Lm* inoculum at “Day 0”, at “Day 6”, and at Day 8, “Day-End”; Table S5: Enumeration of mesophilic aerobic count and *Pseudomonas* spp. in the “Control Unit” samples (inoculated with physiological water in the same volume as inoculum of 10 CFU/g *Lm* in the three Batches of Experiment 1 of raw sea bass fillets; Table S6: Enumeration of mesophilic aerobic count and *Pseudomonas* spp. in the “Test Unit” samples inoculated with 10 CFU/g *Lm* in the three Batches of Experiment 1 of raw sea bass fillets; Table S7: Enumeration of mesophilic aerobic count and *Pseudomonas* spp. in the “Control Unit” samples, inoculated with physiological water in the same volume as inoculum of 50 CFU/g *Lm*, in the three Batches of Experiment 2 of raw sea bass fillets; Table S8: Enumeration of mesophilic aerobic count and *Pseudomonas* spp. in the “Test Unit” samples inoculated with 50 CFU/g *Lm* in the three Batches of Experiment 2 of raw sea bass fillets; Table S9: Enumeration of mesophilic aerobic count and *Pseudomonas* spp. in the “Food Control Samples” of the three Batches of Experiment 1 and Experiment 2 of raw sea bass fillets at “Day 0”
