# Supplementary material for: Assessing Listeria monocytogenes Growth in Artificially Inoculated Sea-Farmed Product—Raw Sea Bass (Dicentrarchus labrax) Fillet, Produced in Greece
Source: Microorganisms. 2024 Sep 28;12(10):1970. doi: 10.3390/microorganisms12101970 (PMC11509366; doi:10.3390/microorganisms12101970)
Supplement: Supplementary file 1 [file microorganisms-12-01970-s001.zip › microorganisms-3182903-supplementary.pdf]

microorganisms

MDPI

Supplementary material

Table S1. Physico-chemical characteristics of the five "Food Control Samples" of each Batch of Experiment 1 (Batch 1,2,3) and Experiment 2 (Batch 4,5,6) of raw sea bass fillets from a sea-farmed product company, produced in Greece.

	Food Control Samples Batch 1				Food Control Samples Batch 2				Food Control Samples Batch 3			
	pH	aw	% NaCl	% Fat	pH	aw	% NaCl	% Fat	pH	aw	% NaCl	% Fat
Experiment 1	6.42	0.984	0.38	9.70	6.54	0.975	0.31	10.5	6.47	0.997	0.38	9.5
	6.56	0.997	0.41	10.20	6.61	0.991	0.45	9.6	6.57	0.986	0.34	8.9
	6.32	0.998	-	-	6.48	0.989	-	-	6.44	0.978	-	-
	6.30	0.975	-	-	6.42	0.979	-	-	6.52	0.985	-	-
	6.50	0.989	-	-	6.63	0.984	-	-	6.46	0.981	-	-
**Average**	**6.42**	**0.989**	**0.40**	**9.95**	**6.54**	**0.984**	**0.38**	**10.05**	**6.49**	**0.985**	**0.36**	**9.20**
**SD**	**0.11**	**0.010**	**0.02**	**0.35**	**0.09**	**0.007**	**0.10**	**0.64**	**0.05**	**0.007**	**0.03**	**0.42**
	Food Control Samples Batch 4				Food Control Samples Batch 5				Food Control Samples Batch 6			
	pH	aw	% NaCl	% Fat	pH	aw	% NaCl	% Fat	pH	aw	% NaCl	% Fat
Experiment 2	6.38	0.979	0.47	8.80	6.45	0.994	0.51	9.4	6.47	0.984	0.42	9.1
	6.62	0.985	0.40	9.60	6.51	0.991	0.40	8.9	6.50	0.979	0.55	9.5
	6.53	0.994	-	-	6.47	0.983	-	-	6.49	0.990	-	-
	6.37	0.977	-	-	6.32	0.995	-	-	6.53	0.971	-	-
	6.55	0.989	-	-	6.50	0.983	-	-	6.50	0.986	-	-
**Average**	**6.49**	**0.985**	**0.44**	**9.20**	**6.45**	**0.989**	**0.46**	**9.15**	**6.50**	**0.982**	**0.49**	**9.30**
**SD**	**0.11**	**0.007**	**0.05**	**0.57**	**0.08**	**0.006**	**0.08**	**0.35**	**0.02**	**0.007**	**0.09**	**0.28**

Table S2. Growth of *Lm* in the "Test Units" of the three Batches of Experiment 1 of raw sea bass fillets from a sea-farmed product company, produced in Greece. The raw sea bass fillets were inoculated with *Lm* 10 CFU/g.

	**Batch 1**	**Batch 2**	**Batch 3**
**"Day 0"**, Enumerate the *Lm* of **Test Unit 1** and store the rest of the Test Units at 2 ^{0}C for 2 days (conditions at manufacture and transport level)	microorganisms are present but <40 CFU/g**	<10 CFU/g*	microorganisms are present but <40 CFU/g**
**"Day 0"**, Enumerate the *Lm* of **Test Unit 2** and store the rest of the Test Units at 2 ^{0}C for 2 days (conditions at manufacture and transport level)	<10 CFU/g*	microorganisms are present but <40 CFU/g**	microorganisms are present but <40 CFU/g**
**"Day 0"**, Enumerate the *Lm* of **Test Unit 3** and store the rest of the Test Units at 2 ^{0}C for 2 days (conditions at manufacture and transport level)	microorganisms are present but <40 CFU/g**	microorganisms are present but <40 CFU/g**	microorganisms are present but <40 CFU/g**
**"Day 2"**, Enumerate the *Lm* of **Test Unit 4** and store the rest of the Test Units at 4 ^{0}C for 2 days (conditions at retail level)	microorganisms are present but <40 CFU/g**	estimated number of microorganisms is 50 CFU/g***	microorganisms are present but <40 CFU/g**
**"Day 4"**, Enumerate the *Lm* of **Test Unit 5** and store the rest of the Test Units at 4 ^{0}C for 2 days (conditions at retail level)	estimated number of microorganisms is 40 CFU/g***	estimated number of microorganisms is 50 CFU/g***	microorganisms are present but <40 CFU/g**
**"Day 6"**, Enumerate the *Lm* of **Test Unit 6** and store the rest of the Test Units at 10 ^{0}C for 2 days (conditions at consumer level)	4.1×10^2 CFU/g	8.3×10^2 CFU/g	2.8×10^2 CFU/g
**Day END, "Day 8",** Enumerate the *Lm* of **Test Unit 7**	3.0×10^3 CFU/g	6.7×10^3 CFU/g	2.1×10^3 CFU/g

Calculations of *Lm* colonies on a plate and expression of the results were performed according to ISO 7218:2007/Amd.1:2013:

* no colonies are observed on the plate (test sample or initial suspension or first dilution was inoculated)

** the total number of *Lm* colonies on the plate is from 1 to 3, the precision of the result is too low, and the result is reported as: "Microorganisms are present but less than 4/Vd per gram or per ml, where 4 represents the minimum number of colonies required for a reliable estimate and Vd is the volume of inoculum placed on each dish, in milliliters, d - is the dilution corresponding to the first dilution retained.

*** the total number of *Lm* colonies on the plate is less than 10 colonies, but at least four; the result is reported as an "estimated number" (a less precise estimate of the true value)

Table S3. Physico-chemical characteristics of the "Control Units" of the three Batches of Experiment 1 of raw sea bass fillets inoculated with physiological water in the same volume as the 10 CFU/g *Lm* inoculum at "Day 0", at "Day 6", and at Day 8, "Day-End".

	**Batch 1 Control Units**				**Batch 2 Control Units**				**Batch 3 Control Units**			
**Day 0** (2°C)	**pH**	**a_w**	**% NaCl**	**% Fat**	**pH**	**a_w**	**% NaCl**	**% Fat**	**pH**	**aw a_w**	**% NaCl**	**% Fat**
1	6,52	0.996	0.35	9.8	6.46	0.989	0.39	9.60	6.57	0.984	0.51	8.60
2	6,44	0.987	0.43	9.6	6.58	0.986	0.36	8.90	6.46	0.992	0.446	10.40
**Average**	**6.48**	**0.99**	**0.39**	**9.7**	**6.52**	**0.99**	**0.375**	**9.25**	**6.515**	**0.988**	**0.478**	**9.50**
**±SD**	**0.06**	**0.01**	**0.06**	**0.14**	**0.08**	**0.002**	**0.021**	**0.495**	**0.078**	**0.006**	**0.045**	**1.273**
**Day 6** (4°C)	**pH**	**a_w**	**% NaCl**	**% Fat**	**pH**	**a_w**	**% NaCl**	**% Fat**	**pH**	**a_w**	**% NaCl**	**% Fat**
1	6,35	0.988	0.36	NE *	6.36	0.985	0.41	NE *	6.39	0.985	0.47	NE *
2	6,26	0.982	0.41	NE *	6.39	0.988	0.38	NE *	6.38	0.984	0.49	NE *
**Average**	**6.305**	**0.99**	**0.39**		**6.375**	**0.987**	**0.395**		**6.385**	**0.985**	**0.48**	
**±SD**	**0.064**	**0.004**	**0.035**		**0.021**	**0.002**	**0.021**		**0.007**	**0.001**	**0.014**	
**Day 8** (10°C)	**pH**	**a_w**	**% NaCl**	**% fat**	**pH**	**a_w**	**% NaCl**	**% fat**	**pH**	**a_w**	**% NaCl**	**% fat**
1	6.12	0.984	0.40	NE *	6.28	0.976	0.35	NE *	6.27	0.979	0.45	NE *
2	6.03	0.979	0.35	NE *	6.31	0.986	0.39	NE *	6.32	0.982	0.51	NE *
**Average**	**6.075**	**0.98**	**0.38**		**6.295**	**0.981**	**0.37**		**6.295**	**0.981**	**0.48**	
**±SD**	**0.064**	**0.004**	**0.035**		**0.021**	**0.007**	**0.028**		**0.035**	**0.002**	**0.042**	

*not examined

Table S4. Physico-chemical characteristics of the "Control Units" of the three Batches of Experiment 2 of raw sea bass fillets inoculated with physiological water in the same volume as the 50 CFU/g *Lm* inoculum at "Day 0", at "Day 6", and at Day 8, "Day-End".

	**Batch 4 Control Units**				**Batch 5 Control Units**				**Batch 6 Control Units**			
**Day 0** (2°C)	**pH**	**a_w**	**% NaCl**	**% Fat**	**pH**	**a_w**	**% NaCl**	**% Fat**	**pH**	**a_w**	**% NaCl**	**% Fat**
1	6,63	0.993	0.48	8.9	6.47	0.989	0.47	8.50	6.50	0.992	0.53	8.70
2	6,48	0.984	0.50	9.5	6.52	0.988	0.40	10.20	6.36	0.987	0.42	8.30
**Average**	**6.555**	**0.99**	**0.49**	**9.2**	**6.495**	**0.989**	**0.435**	**9.350**	**6.43**	**0.990**	**0.475**	**8.50**
**±SD**	**0.106**	**0.01**	**0.014**	**0.424**	**0.035**	**0.001**	**0.049**	**1.202**	**0.099**	**0.004**	**0.078**	**0.283**
**Day 6** (4°C)	**pH**	**a_w**	**% NaCl**	**% Fat**	**pH**	**a_w**	**% NaCl**	**% Fat**	**pH**	**a_w**	**% NaCl**	**% Fat**
1	6,25	0.984	0.37	NE *	6.16	0.983	0.45	NE *	6.24	0.988	0.51	NE *
2	6,19	0.979	0.42	NE *	6.36	0.987	0.37	NE *	6.29	0.984	0.45	NE *
**Average**	**6.22**	**0.98**	**0.395**		**6.26**	**0.985**	**0.41**		**6.265**	**0.986**	**0.48**	
**±SD**	**0.042**	**0.004**	**0.035**		**0.141**	**0.003**	**0.057**		**0.035**	**0.003**	**0.042**	
**Day 8** (10°C)	**pH**	**a_w**	**% NaCl**	**% fat**	**pH**	**a_w**	**% NaCl**	**% fat**	**pH**	**a_w**	**% NaCl**	**% fat**
1	6.17	0.978	0.43	NE *	6.03	0.977	0.50	NE *	6.11	0.978	0.42	NE *
2	6.06	0.984	0.35	NE *	6.10	0.982	0.43	NE *	6.05	0.984	0.47	NE *
**Average**	**6.115**	**0.981**	**0.39**		**6.065**	**0.980**	**0.465**		**6.08**	**0.981**	**0.445**	
**±SD**	**0.078**	**0.004**	**0.057**		**0.049**	**0.004**	**0.049**		**0.042**	**0.004**	**0.035**	

*not examined

Table S5. Enumeration of mesophilic aerobic count and *Pseudomonas* spp. in the "Control Unit" samples (inoculated with physiological water in the same volume as inoculum of 10 CFU/g *Lm* in the three Batches of Experiment 1 of raw sea bass fillets.

	Mesophilic aerobic count (log_{10} CFU/g)			*Pseudomonas* spp. (log_{10} CFU/g)		
**Day**	**Batch 1**	**Batch 2**	**Batch 3**	**Batch 1**	**Batch 2**	**Batch 3**
0 (2^{0}C) "Day 0"	3.44	3.66	3.06	3.32	3.63	3.04
4 (4^{0}C)	5.82	6.12	5.45	5.67	5.94	5.34
6 (4^{0}C)	7.55	7.98	7.42	7.53	7.95	7.28
8 (10^{0}C) "Day End"	9.26	9.32	8.99	9.54	9.34	8.88
% concentration increase from "Day 0" to "Day 4"	69.28	67.33	77.98	70.66	63.63	75.77
% concentration increase from "Day 4" to "Day 6"	29.81	30.46	36.17	32.84	33.74	36.38
% concentration increase from "Day 6" to "Day 8"	22.68	16.79	21.17	26.73	17.54	22.00
% concentration increase from "Day 0" to "Day 8"	169.58	154.93	193.65	187.29	157.22	192.45

Table S6. Enumeration of mesophilic aerobic count and *Pseudomonas* spp. in the "Test Unit" samples inoculated with 10 CFU/g *Lm* in the three Batches of Experiment 1 of raw sea bass fillets.

	Mesophilic aerobic count (log_{10} CFU/g)			*Pseudomonas* spp. (log_{10} CFU/g)		
**Day**	**Batch 1**	**Batch 2**	**Batch 3**	**Batch 1**	**Batch 2**	**Batch 3**
0 (2^{0}C) "Day 0"	3.48	3.71	3.19	3.42	3.68	3.13
4 (4^{0}C)	5.93	6.18	5.77	5.87	6.10	5.59
6 (4^{0}C)	7.93	8.10	7.76	7.87	8.08	7.65
8 (10^{0}C) "Day End"	9.20	9.51	9.10	9.21	9.53	9.11
% concentration increase from "Day 0" to "Day 4"	70.59	66.27	80.99	71.66	65.59	78.40
% concentration increase from "Day 4" to "Day 6"	33.72	31.08	34.42	33.97	32.42	36.78
% concentration increase from "Day 6" to "Day 8"	16.01	17.54	17.24	17.12	17.97	19.12
% concentration increase from "Day 0" to "Day 8"	164.63	156.16	185.22	169.34	158.68	190.67

Table S7. Enumeration of mesophilic aerobic count and *Pseudomonas* spp. in the "Control Unit" samples (inoculated with physiological water in the same volume as inoculum of 50 CFU/g *Lm*) in the three Batches of Experiment 2 of raw sea bass fillets.

	**Mesophilic aerobic count (log_{10} CFU/g)**			***Pseudomonas* spp. (log_{10} CFU/g)**		
**Day**	**Batch 4**	**Batch 5**	**Batch 6**	**Batch 4**	**Batch 5**	**Batch 6**
0 (2^{0}C) "Day 0"	3.73	3.88	3.56	3.46	3.74	3.39
4 (4^{0}C)	5.99	6.19	5.67	5.99	6.07	5.55
6 (4^{0}C)	8.06	8.28	7.65	8.21	8.37	7.79
8 (10^{0}C) "Day End"	9.15	9.40	8.93	9.61	9.58	9.13
% concentration increase from "Day 0" to "Day 4"	60.67	59.54	59.37	72.88	62.33	63.71
% concentration increase from "Day 4" to "Day 6"	34.55	33.82	34.79	37.17	38.05	40.39
% concentration increase from "Day 6" to "Day 8"	13.51	13.54	16.77	17.02	14.43	17.24
% concentration increase from "Day 0" to "Day 8"	145.39	142.40	150.85	177.50	156.42	169.47

Table S8. Enumeration of mesophilic aerobic count and *Pseudomonas* spp. in the "Test Unit" samples inoculated with 50 CFU/g *Lm* in the three Batches of Experiment 2 of raw sea bass fillets.

	**Mesophilic aerobic count (log_{10} CFU/g)**			***Pseudomonas* spp. (log_{10} CFU/g)**		
**Day**	**Batch 4**	**Batch 5**	**Batch 6**	**Batch 4**	**Batch 5**	**Batch 6**
0 (2^{0}C) Day 0	3.87	3.93	3.67	3.61	3.86	3.45
4 (4^{0}C)	6.23	6.66	5.95	6.10	6.28	5.94
6 (4^{0}C)	8.36	8.92	8.00	8.28	8.76	7.97
8 (10^{0}C) "Day End"	9.75	9.94	9.40	9.67	9.84	9.39
% concentration increase from "Day 0" to "Day 4"	60.83	69.53	61.92	68.85	62.88	72.07
% concentration increase from "Day 4" to "Day 6"	34.20	34.02	34.39	35.78	39.55	34.21
% concentration increase from "Day 6" to "Day 8"	16.66	11.36	17.55	16.83	12.26	17.86
% concentration increase from "Day 0" to "Day 8"	151.81	153.02	155.80	167.87	155.16	172.18

Table S9: Enumeration of mesophilic aerobic count and *Pseudomonas* spp. in the "Food Control Samples" of the three Batches of Experiment 1 and Experiment 2 of raw sea bass fillets at "Day 0".

	**Mesophilic aerobic count (log_{10} CFU/g)**			***Pseudomonas* spp. (log_{10} CFU/g)**		
	**Batch 1**	**Batch 2**	**Batch 3**	**Batch 1**	**Batch 2**	**Batch 3**
(2^0C) "Day 0", Experiment 1	3.30	3.09	3.42	3.37	3.10	3.21
	**Batch 4**	**Batch 5**	**Batch 6**	**Batch 4**	**Batch 5**	**Batch 6**
(2^0C) "Day 0", Experiment 2	3.35	3.70	3.45	3.42	3.28	3.73
